# Supplementary material for: Resource utilization and outcomes in emergency general surgery during the COVID19 pandemic: An observational cost analysis
Source: PLoS One. 2021 Jun 18;16(6):e0252919. doi: 10.1371/journal.pone.0252919 (PMC8213049; doi:10.1371/journal.pone.0252919)

PLOS ONE

RESOURCE UTILIZATION AND OUTCOMES IN EMERGENCY GENERAL SURGERY DURING THE COVID19 PANDEMIC: AN OBSERVATIONAL COST ANALYSIS

--Manuscript Draft--

**Manuscript Number:**	PONE-D-21-12253
**Article Type:**	Research Article
**Full Title:**	RESOURCE UTILIZATION AND OUTCOMES IN EMERGENCY GENERAL SURGERY DURING THE COVID19 PANDEMIC: AN OBSERVATIONAL COST ANALYSIS
**Short Title:**	RESOURCE UTILIZATION AND COSTS IN EMERGENCY SURGERY DURING COVID19
**Corresponding Author:**	Amelia Hessheimer<br>Hospital Clinic de Barcelona<br>Barcelona, Barcelona SPAIN
**Keywords:**	COVID19; emergency general surgery; acute care surgery; cost analysis
**Abstract:**	Background: Over the course of the COVID19 pandemic, global healthcare delivery has declined. Surgery is one of the most resource-intensive area of medicine; loss of surgical care has had untold health and economic consequences. Herein, we evaluate resource utilization, outcomes, and direct healthcare costs associated with unplanned surgery admissions during the height of the pandemic in 2020 versus the same period in 2019.<br>Methods: Retrospective analysis on patients ≥18 years admitted from the emergency department to General & Digestive and Gastrointestinal Surgery Services between February and May 2019 and 2020 at Hospital Clínic Barcelona; clinical outcomes and unadjusted and adjusted per-person healthcare costs were analyzed.<br>Results: Consults and admissions to surgery declined between February and May 2020 by 37% and 19%, respectively, relative to the same period in 2019, with even greater relative decline during late March and early April. Time between onset of symptoms to diagnosis increased from 2±3 days 2019 to 5±22 days 2020 (P =0.01). Overall hospital stay was two days less in 2020 (P =0.19). Complications (Comprehensive Complication Index 10.3±23.7 2019 vs. 13.9±25.5 2020, P =0.10) and mortality rates (3% vs. 4%, respectively, P =0.58) did not vary. Mean unadjusted per-person costs for patients in the 2019 and 2020 cohorts were 5,886.72€±12,576.33€ and 5,287.62±7,220.16€, respectively (P =0.43). Following multivariate analysis, costs remained similar (4,656.89€±390.53€ 2019 vs. 4,938.54±406.55€ 2020, P =0.28)<br>Conclusions: Healthcare delivery and spending for unplanned general surgery admissions declined considerably due to COVID19. These results provide a small yet relevant illustration of clinical and economic ramifications of this healthcare crisis.
**Order of Authors:**	Amelia Hessheimer
	Marta Trapero
	Alex Borin
	Eugenia Butori
	Anna Curell
	Arlena Sofía Espinoza
	Joaquín Jensen
	Víctor Turrado
	Xavier Morales
	Antonio María de Lacy
	Constantino Fondevila

Powered by Editorial Manager® and ProduXion Manager® from Aries Systems Corporation

Additional Information:	
**Question**	**Response**
**Financial Disclosure**<br><br>Enter a financial disclosure statement that describes the sources of funding for the work included in this submission. Review the submission guidelines for detailed requirements. View published research articles from *PLOS ONE* for specific examples.<br><br>This statement is required for submission and **will appear in the published article** if the submission is accepted. Please make sure it is accurate.<br><br>**Unfunded studies**<br>Enter: *The author(s) received no specific funding for this work.*<br><br>**Funded studies**<br>Enter a statement with the following details:<br>• Initials of the authors who received each award<br>• Grant numbers awarded to each author<br>• The full name of each funder<br>• URL of each funder website<br>• Did the sponsors or funders play any role in the study design, data collection and analysis, decision to publish, or preparation of the manuscript?<br>• **NO** - Include this sentence at the end of your statement: *The funders had no role in study design, data collection and analysis, decision to publish, or preparation of the manuscript.*<br>• **YES** - Specify the role(s) played.<br><br>* typeset	AJH and CF have been funded by FIS Grant PI18-00894 from the Instituto de Salud Carlos III (ISCIII). The funders had no role in study design, data collection and analysis, decision to publish, or preparation of the manuscript.
**Competing Interests**<br><br>Use the instructions below to enter a competing interest statement for this submission. On behalf of all authors, disclose any competing interests that could be perceived to bias this work—acknowledging all financial support and any other relevant financial or non-financial competing interests.	AJH and CF have received consultancy fees from Guanguong Shunde Innovative Design Institute, Guangdong, China. The remainder of the authors have no disclosures.

This statement is required for submission and **will appear in the published article** if the submission is accepted. Please make sure it is accurate and that any funding sources listed in your Funding Information later in the submission form are also declared in your Financial Disclosure statement.<br><br>View published research articles from *PLOS ONE* for specific examples.<br><br>**NO authors have competing interests**<br><br>Enter: *The authors have declared that no competing interests exist.*<br><br>**Authors with competing interests**<br><br>Enter competing interest details beginning with this statement:<br><br>*I have read the journal's policy and the authors of this manuscript have the following competing interests: [insert competing interests here]*<br><br>* typeset	
**Ethics Statement**<br><br>Enter an ethics statement for this submission. This statement is required if the study involved:<br><br>• Human participants<br>• Human specimens or tissue<br>• Vertebrate animals or cephalopods<br>• Vertebrate embryos or tissues<br>• Field research<br><br>Write "N/A" if the submission does not require an ethics statement.<br><br>General guidance is provided below. Consult the submission guidelines for detailed instructions. **Make sure that all information entered here is included in the**	Study approval was obtained from the HCB Committee on Ethics in Medical Research prior to data collection (study number HCB/2020/0542), which waived need for written consent.

**Methods section of the manuscript.**<br><br>**Format for specific study types**<br><br>**Human Subject Research (involving human participants and/or tissue)**<br>• Give the name of the institutional review board or ethics committee that approved the study<br>• Include the approval number and/or a statement indicating approval of this research<br>• Indicate the form of consent obtained (written/oral) or the reason that consent was not obtained (e.g. the data were analyzed anonymously)<br><br>**Animal Research (involving vertebrate animals, embryos or tissues)**<br>• Provide the name of the Institutional Animal Care and Use Committee (IACUC) or other relevant ethics board that reviewed the study protocol, and indicate whether they approved this research or granted a formal waiver of ethical approval<br>• Include an approval number if one was obtained<br>• If the study involved *non-human primates*, add *additional details* about animal welfare and steps taken to ameliorate suffering<br>• If anesthesia, euthanasia, or any kind of animal sacrifice is part of the study, include briefly which substances and/or methods were applied<br><br>**Field Research**<br><br>Include the following details if this study involves the collection of plant, animal, or other materials from a natural setting:<br>• Field permit number<br>• Name of the institution or relevant body that granted permission	
**Data Availability**<br><br>Authors are required to make all data underlying the findings described fully available, without restriction, and from the time of publication. PLOS allows rare exceptions to address legal and ethical concerns. See the PLOS Data Policy and FAQ for detailed information.	Yes - all data are fully available without restriction

Powered by Editorial Manager® and ProduXion Manager® from Aries Systems Corporation

A Data Availability Statement describing where the data can be found is required at submission. Your answers to this question constitute the Data Availability Statement and **will be published in the article**, if accepted.<br><br>**Important:** Stating 'data available on request from the author' is not sufficient. If your data are only available upon request, select 'No' for the first question and explain your exceptional situation in the text box.<br><br>Do the authors confirm that all data underlying the findings described in their manuscript are fully available without restriction?	
**Describe where the data may be found in full sentences. If you are copying our sample text, replace any instances of XXX with the appropriate details.**<br><br>• If the data are **held or will be held in a public repository**, include URLs, accession numbers or DOIs. If this information will only be available after acceptance, indicate this by ticking the box below. For example: *All XXX files are available from the XXX database (accession number(s) XXX, XXX.*).<br>• If the data are all contained **within the manuscript and/or Supporting Information files**, enter the following: *All relevant data are within the manuscript and its Supporting Information files.*<br>• If neither of these applies but you are able to provide **details of access elsewhere**, with or without limitations, please do so. For example:<br><br>*Data cannot be shared publicly because of [XXX]. Data are available from the XXX Institutional Data Access / Ethics Committee (contact via XXX) for researchers who meet the criteria for access to confidential data.*<br><br>*The data underlying the results*	Data cannot be shared publicly. Data are available from the Hospital Clínic Institutional Data Access / Ethics Committee (contact via Amelia J. Hessheimer) for researchers who meet the criteria for access to confidential data.

Powered by Editorial Manager® and ProduXion Manager® from Aries Systems Corporation

presented in the study are available from (include the name of the third party and contact information or URL).<br>• This text is appropriate if the data are owned by a third party and authors do not have permission to share the data.<br><br>* typeset	
Additional data availability information:	

Powered by Editorial Manager® and ProduXion Manager® from Aries Systems Corporation

Manuscript

Click here to access/download;Manuscript;MANUSCRIPT_COSTS_COVID

RESOURCE UTILIZATION AND OUTCOMES IN EMERGENCY GENERAL SURGERY DURING THE COVID19 PANDEMIC: AN OBSERVATIONAL COST ANALYSIS

Amelia J. Hessheimer, MD, PhD[1], Marta Trapero-Bertran, PhD[2], Alex Borin, MD[3], Eugenia Butori, MD[3,4], Anna Curell, MD[4], Arlena Sofía Espinoza, MD[3,4], Joaquín Jensen, MD[3], Víctor Turrado, MD[4], Xavier Morales, MD[4], Antonio María de Lacy, MD, PhD[4], and Constantino Fondevila, MD, PhD[1]

[1]General & Digestive Surgery, Institut de Malaties Digestives i Metabòliques (ICMDM), Hospital Clínic Barcelona, IDIBAPS, CIBERehd, University of Barcelona, Barcelona, Spain

[2]Basic Sciences Department, University Institute for Patient Care, Universitat Internacional de Catalunya Barcelona, Barcelona, Spain

[3] General & Digestive Surgery, ICMDM, Hospital Clínic Barcelona, Barcelona, Spain

[4]Gastrointestinal Surgery, ICMDM, Hospital Clínic Barcelona, Barcelona, Spain

CORRESPONDING AUTHOR INFORMATION:

Amelia J. Hessheimer, MD, PhD
General & Digestive Surgery, Hospital Clínic
C/ Villarroel 170
08036 Barcelona, Spain
Phone: +34 93 227 57 18
Email: AJHESSHE@clinic.cat

ABSTRACT

Background: Over the course of the COVID19 pandemic, global healthcare delivery has declined. Surgery is one of the most resource-intensive area of medicine; loss of surgical care has had untold health and economic consequences. Herein, we evaluate resource utilization, outcomes, and direct healthcare costs associated with unplanned surgery admissions during the height of the pandemic in 2020 versus the same period in 2019.

Methods: Retrospective analysis on patients ≥18 years admitted from the emergency department to General & Digestive and Gastrointestinal Surgery Services between February and May 2019 and 2020 at Hospital Clínic Barcelona; clinical outcomes and unadjusted and adjusted per-person healthcare costs were analyzed.

Results: Consults and admissions to surgery declined between February and May 2020 by 37% and 19%, respectively, relative to the same period in 2019, with even greater relative decline during late March and early April. Time between onset of symptoms to diagnosis increased from 2±3 days 2019 to 5±22 days 2020 (P=0.01). Overall hospital stay was two days less in 2020 (P=0.19). Complications (Comprehensive Complication Index 10.3±23.7 2019 vs. 13.9±25.5 2020, P=0.10) and mortality rates (3% vs. 4%, respectively, P=0.58) did not vary. Mean unadjusted per-person costs for patients in the 2019 and 2020 cohorts were 5,886.72€±12,576.33€ and 5,287.62±7,220.16€, respectively (P=0.43). Following multivariate analysis, costs remained similar (4,656.89€±390.53€ 2019 vs. 4,938.54±406.55€ 2020, P=0.28)

Conclusions: Healthcare delivery and spending for unplanned general surgery admissions declined considerably due to COVID19. These results provide a small yet relevant illustration of clinical and economic ramifications of this healthcare crisis.

KEYWORDS: COVID19, emergency general surgery, acute care surgery, cost analysis.

ABBREVIATIONS: AIC, Akaike information criterion; ASA, American Society of Anesthesiologists; CCI, Comprehensive Complication Index; ED, emergency department; GLM, generalized linear models; HCB, Hospital Clínic Barcelona; ICU, intensive care unit; OR, operating room.

INTRODUCTION

Apart from its direct impact on infected patients, the SARS-CoV-2/COVID19 pandemic has had indirect collateral effects on patients with other pathologies who have accessed standard of care for their diseases[1,2]. Surgery is one of the most resource-intensive areas of clinical medicine[3], and patients requiring surgical interventions have been particularly vulnerable in this regard. Factors adversely affecting surgical patients have included lack of operating room personnel and space. Inability and/or fear of patients with incidental surgical pathology to present in timely fashion has resulted in a well-documented declines in surgical consults during the pandemic in many parts of the world[4–9]. Moreover, it has been suggested that these patients may have also presented later, with more advanced disease[9,10]. These issues have potential to not only impact patients' lives and quality of life but also put further strain on public money and healthcare resources at a moment when resources are severely diminished.

Cost studies in healthcare are performed with the objective of transforming the impact of a disease in economic terms. They provide empirical evidence to facilitate assessment by public decision-makers, who have limited budgets, in order to make rational decisions regarding efficient allocation of resources.

The degree to which patients with incidental surgical pathology derived from the emergency department may have been associated with a change in direct healthcare spending during the COVID19 pandemic is not well understood. We hypothesized that analysis of costs associated with unplanned general surgery admissions during 2020 would likely demonstrate differences versus those of the previous year, 2019, where no pandemic occurred. We performed an exploratory cost analysis to determine differences in resource utilization, clinical outcomes, and direct healthcare costs, aiming to identify what patterns may have existed and if they varied according to presenting diagnosis, in order to identify any potential areas for improving healthcare expenditures without compromising patient outcomes in future waves of the same disease or new pandemics of a similar nature.

PATIENTS & METHODS

Study design

This is a retrospective study on adult patients (≥18 years) admitted from the Emergency Department (ED) to the General & Digestive and Gastrointestinal Surgery Services at the Hospital Clínic Barcelona (HCB), a tertiary care center serving an encatchment population of 540,000. Patients admitted between February and May 2020 and the same period in 2019 were included.

Data was collected from the HCB Electronic Medical Record (SAP, S.E., Walldorf, Baden-Württemberg, Germany) and the Shared Medical Record of the Catalan Department of Health. Data collection did not violate any of the usual data provider processes. This study was conducted in compliance with the Declaration of Helsinki (current version October 2013, Fortaleza, Brazil) and in accordance with existing protocol and legal requirements ("Boletín Oficial del Estado Orden SAS/3470/2009", December 16, 2009). Study approval was obtained from the HCB Committee on Ethics in Medical Research prior to data collection (study number HCB/2020/0542), which waived need for written consent.

Definition of variables and outcomes

The main objectives of the study were to assess clinical outcomes and costs associated with general surgery ED admissions during the peak of the pandemic in 2020 relative to the same period in 2019. The study was conducted from the perspective of the hospital provider; no primary care or other ambulatory healthcare costs were imputed.

Clinical variables and outcomes. The following baseline sociodemographic and clinical variables were recorded: age, sex, country of birth, civil status, level of education, profession, employment situation, American Society of Anesthesiologists (ASA) classification[11], and history of previous surgery. Variables related to admission included principal diagnosis; date of diagnosis; duration of symptoms prior to diagnosis; and non-surgical treatment, when applicable, and success or failure of such treatment. Surgical variables, when applicable, included date of surgery, intraoperative complications, and operative time. Hospitalization variables included length of stay and, when applicable, intensive care unit (ICU) stay; surgical

reintervention; mortality during index admission or up to 30 days thereafter; and the Comprehensive Complication Index[12], which computes all complications a patient experiences in an index ranging from 0 to 100. Diagnosis of COVID19 was recorded for patients in the 2020 cohort.

Admissions were grouped into the following diagnostic categories: appendicitis (acute forms, including appendicular infiltrate), biliary tract pathology (acute cholecystitis, cholangitis, choledocholithiasis, and other forms of obstructive jaundice), gastrointestinal obstruction (partial and complete obstruction due to adhesive disease, herniation, neoplasm, volvulus, etc.), non-obstructive gastrointestinal pathology (including acute diverticulitis, colitis, and gastrointestinal tract perforation of any cause), skin and soft tissue infection (including perianal and other superficial abscesses and necrotizing fasciitis), abdominal trauma (both blunt and penetrating), and readmission (complications arising as a direct consequence of a previous surgical intervention or within 30 days of discharge). Admissions falling outside these categories were categorized as other.

Costs. All costs are expressed in 2020 Euros (€). Costs associated with index admission and readmissions with 30 days of discharge were obtained from the Hospital Clínic. Costs of hospitalization included pharmacy, transfusions, artificial implants (stent, mesh, etc.), miscellaneous consumable materials, physician medical care, general floor stay, studies and procedures (radiological, endoscopic, etc.), laboratory tests, consultations from other specialists (physiotherapy, nutritional services), and sanitation/janitorial services. Operative costs included consumables (anesthetic drugs, transfusions, surgical instrumentation, and devices) and operating room (OR) and staff costs. Death did not result in cost-censoring and was considered to represent complete data in that no further cost was accrued; costs of patients who died before discharge were included in the analysis.

Data presentation and analysis

Unadjusted and adjusted direct healthcare costs are presented for the four-month periods in 2019 and 2020. Continuous variables are presented as arithmetic mean ± standard deviation and categorical variables as frequencies, unless otherwise specified. Univariate analysis of costs and

differences in continuous and categorical variables were assessed using the parametric test of cost on untransformed (raw) scale, univariate two-sample *t* test, and Chi-squared test, respectively. Multivariate analysis of costs was performed to adjust for potential cost variation due to other causes (confounding variables). Generalized linear models (GLM) were used for multivariate analysis, as they allow for heteroscedasticity through a variance structure, relating variance to mean[13]. The modified Parks test was used to identify the Gamma family for the GLM[14]. In certain cost categories where a large number of cases incurred no costs, a two-part model was used. The two-part model consisted in a probit model followed by GLM, with Gamma family and log link. To select the model with the best fit, we evaluated Pseudo R2 for the first part and the Akaike information criterion (AIC) for the second part of the model. Statistical significance was defined as $P<0.05$. Statistical analyses were performed using STATA® 16.0 (StataCorp, LLC, College Station, Texas, USA).

RESULTS

In the four-month periods in 2019 and 2020, there were a total of 33,654 and 23,627 consults to the HCB Emergency Department, among which 4,326 (13%) and 2,722 (12%), respectively, were triaged to general surgery (59% decrease between years). From these populations, 772 distinct patients were ultimately admitted and included in the analysis: 426 from 02-05/2019 and 346 from 02-05/2020 (23% decreased between years).

General surgery consults and admissions from the ED were relatively stable throughout the four-month period in 2019, with higher activity in March relative to the other three months. While consults and admissions were similar in February 2019 and 2020, both declined considerably in March 2020 relative to both March 2019 (consults -55%, admissions -48%) and February 2020 (consults -48%, admissions -41%). This decline continued through April and returned to near normal by May 2020 ***(FIGURE 1)***.

Patient demographics

Baseline patient sociodemographic and clinical characteristics are listed in ***TABLE 1***. The average age of all patients was 60±20 years, 43% were women, 78% were from Spain, and 39%

were married; 46% were pensioners who had previously worked and another 37% were actively employed. Age, sex, country of birth, employment situation, profession, ASA classification, and history of prior surgery did not differ between the two cohorts; civil status was unavailable for a greater proportion of case in the 2020 cohort relative to 2019.

Clinical variables and outcomes

TABLE 2 provides data from the two cohorts related to admissions, interventions, and overall hospital stays. Among patients in the 2020 cohort, ten (3%) were concomitantly diagnosed with COVID19.

There were differences in the representation of the different diagnostic categories between the two periods, with the greatest number of admissions in 2019 related to acute appendicitis (N=106, 25%) and the greatest number in 2020 related to biliary tract pathology (N=103, 30%). Time between onset of symptoms to diagnosis was significantly increased from an average of two days in 2019 to an average of five days in 2020 (P=0.01). The percent of cases initially managed non-operatively and rate of failure of non-operative management did not differ between the two periods. Intraoperative complications and operative times were similar. Overall hospital stay was, on average, two days less in 2020, though this difference was not statistically significant. Complications (CCI 10.3±23.7 2019 group vs. 13.9±25.5 2020 group, P=0.10) and mortality rates (3% vs. 4%, respectively, P=0.58) did not vary.

Cost outcomes

Costs for hospitalization, surgery, and readmissions were available for 409 patients from 2019 and 334 from 2020.

Overall costs outcomes. Mean unadjusted per-person healthcare costs for patients in the 2019 and 2020 cohorts were 5,886.72€±12,576.33€ and 5,287.62±7,220.16€, respectively (P=0.43). Median per-person costs were lower in both instances (2,532.77€ and 3,217.40€, respectively), indicating that cost distributions were skewed to the left and the majority of patients actually had costs below the arithmetic means. ***FIGURE 2*** reflects how costs were distributed per month in each period.

Following unadjusted univariate analysis, a few significant differences between the two periods were noted, with higher costs associated with miscellaneous consumables used during hospitalization and lower costs associated with OR consumables and OR usage in 2020 relative to 2019 ***(TABLE 3)***. Following multivariate analysis and adjustment of costs to account for potential influence of confounding covariates, mean per-person healthcare costs remained similar between the two periods. Marginal but statistically significant differences were observed for costs associated with pharmacy and miscellaneous consumable materials (higher in 2020) and OR consumables (lower in 2020).

Outcomes according to diagnosis. In the three largest diagnostic categories – appendicitis, biliary tract pathology, and gastrointestinal obstruction – there were a total of 103, 87, and 69 patients, respectively, with costs available from 2019 and 71, 101, and 45, respectively, from 2020. While 2019 and 2020 costs were the same in the latter two categories, univariate analysis reflected differences in unadjusted costs in the appendicitis subpopulation, with higher total healthcare costs in 2020 relative to 2019 (3,324.93€±175.80€ vs. 2,663.76€±96.49€, respectively, $P<0.01$). Cost differences were based in large part on higher costs for general floor stay (829.01€±78.37€ vs. 636.15€±51.55€, $P=0.03$), physician medical care (160.68€±18.13€ vs. 102.81€±9.89€, $P<0.01$), miscellaneous consumable materials (164.65€±33.42€ vs. 2.84€±2.84€, $P<0.01$), and OR staff (688.51€±37.07€ vs. 562.27€±25.49€, $P<0.01$). Given relatively small sample sizes and missing data among covariates, cost adjustments could not be performed. Of note, comprehensive complications were no higher in appendicitis patients treated in 2020 relative to 2019 (CCI 11.9±24.8 vs. 11.7±24.5, respectively).

DISCUSSION

The World Health Organization declared SARS-CoV-2 a global pandemic on March 11, 2020; national lockdown in Spain was implemented three days later. At our center, ED consults and admissions for COVID19 infection rose progressively until they peaked the last week in March. Coincidentally, consults for other urgent pathology declined considerably, and overall ED volume was reduced[5]. The peak was associated with >80% occupation of intensive care and

50% occupation of general floor beds with COVID19 patients. As of this writing, late March and early April continue to represent the maximum point of COVID19 hospitalizations in our region and center.

The objective of this study was not to evaluate costs specifically associated with COVID19 admissions but, rather, those associated with other urgent disease processes displaced or delayed – for whatever reason – by COVID19. Based on this study's findings, in spite of the fact that ED consults and admissions to surgery declined by up to nearly 50% relative to the same period in 2019 and presentations were delayed by an average of three days, mean per-patient costs of hospitalization (including readmissions in addition to index admission) were no different during the peak of the pandemic relative to the same period in 2019.

A logical assumption is that patients arriving later would have arrived sicker. The concept of fewer but sicker patients has not been borne out in this study in terms of the complications, mortality, and costs we observed. Costs have been seen to be significantly associated with the number and severity of complications arising in the course of a disease process[15,16]. Fewer general surgery patients were attended and admitted from the ED between February and May 2020 relative to the same period in 2019, but those that were seen in 2020 did not present higher level of acuity, on average, than those seen in the previous period without pandemic.

We considered that while average costs might have been globally the same for the four-month periods under evaluation, a more detailed analysis comparing individual months might reveal differences. As reflected in ***FIGURE 2***, March 2020 was the month with the lowest per-person healthcare costs. The decline in March, however, was not followed by any increase in costs in April or, for that matter, May 2020. That is to say, there was no rebound effect in the opposite direction following the initial reduction in usage of emergency general surgery services.

While total per-person costs did not vary between the two periods under evaluation, some marginal but nonetheless significant differences were observed in adjusted costs associated with consumable materials, in particular. While small, what these differences likely reflect, if anything, are changes in practice patterns in 2020 relative to 2019 (using, for example, more

personal protective equipment in 2020) as opposed to higher degree of medical or surgical acuity, as clinical outcomes did not vary.

Considering the diagnostic subpopulations, admissions for biliary tract pathology were similar if not slightly higher in 2020 relative to 2019. This observation appears consistent with what is known about dietary and other lifestyle changes that occur(ed) during the pandemic that are largely known to exacerbate biliary tract disease[17–19]. It may be the case that there was some decline in "appendicitis" cases based on fewer presentations of right lower quadrant pain that ultimately resolved with symptomatic/medical management. Given that this study only includes costs associated with hospitalization and not outpatient costs, it is plausible that reduction in ED services for other diagnostic subsets, including obstructive and non-obstructive gastrointestinal pathology (perforations, inflammatory pathology, symptomatic tumors, etc.), was compensated to some degree by a shift toward greater utilization of outpatient resources. Outpatient care, however, appears to have experienced a similar decline as urgent/emergent care consults, and healthcare in general was lost during the height of the pandemic in various parts of the world[1,20,21]. Whether loss of healthcare for non-COVID19 patients accounts for some proportion of excess mortality observed in different countries, including Spain, where excess mortality between February and May was 38%[22], is conceivable but difficult to assess at this point.

We attempted to evaluate cost differences in different diagnostic subpopulations. It is unclear whether cost differences observed for the appendicitis subpopulation, with higher apparent costs in 2020 relative to 2019 on univariate analysis, were truly related to period or were a consequence of confounding variables, as cost adjustments could not be performed. The fact that comprehensive complications for appendicitis admissions were no different in 2020 relative to 2019 is more indicative of the latter.

Based on the reduction in unplanned general surgery admissions and lack of simultaneous increase in per-patient costs for hospitalization, we estimate that healthcare spending at our center was reduced by nearly 650,000€ for this particular patient population between February and May 2020 relative 2019. General surgery is responsible for 12-13% of our emergency department volume, not to mention elective activity, and we can extrapolate further by

considering the sum of similarly resource-intensive areas of medicine and reasonably assume that millions of Euros – potentially in excess of 5-6 million – in healthcare payments were lost at our institution during the height of the pandemic, unrelated to the actual treatment of COVID19. The ultimate repercussions of such findings depend on the healthcare structure and reimbursement system but may be considered to include everything from risk of healthcare-related job losses and loss of income for third parties providing healthcare-related goods and services to considerable financial profits for healthcare insurance providers due to unspent premiums[23].

This study has several limitations. Results we present are from the first wave of the pandemic in Spain and are influenced by the specific lockdown measures that were implemented at that time. As well, it considers resource utilization and costs in emergency general surgery only and does not evaluate other areas of healthcare. A study from Asia examined orthopedic surgery charges and found similar if not reduced in-hospital costs for orthopedic procedures (both urgent and elective) during the pandemic relative to pre-pandemic[24]. Another study from China regarding acute ischemic stroke, however, observed the opposite effect, with higher hospitalization costs during the height of the pandemic there[25]. Based on these findings, further cost studies in other areas of clinical medicine, in particular those requiring direct patient intervention and other activities that cannot be completed via telemedicine, are encouraged.

CONCLUSIONS

During the height of the first wave of the COVID19 pandemic, in spite of up to an almost 50% reduction in emergency general surgery consults and admissions, no differences in outcomes or per-patient hospital-associated healthcare costs were observed at our center. It is improbable that pathology truly disappeared, and this decline in unplanned surgical activity undoubtedly represents part of the "untold burden" of patients that failed to seek care. As the pandemic progresses, it is important to continue to document not only the health-related effects but also the economic consequences of loss of healthcare, as they have important implications for patients, providers, payers, other healthcare stakeholders, and society.

DECLARATIONS

ETHICS APPROVAL AND CONSENT TO PARTICIPATE: Study approval was obtained from the HCB Committee on Ethics in Medical Research prior to data collection (study number HCB/2020/0542), which waived need for written consent.

CONSENT FOR PUBLICATION: The present study does not include details, images, or videos relating to an individual person.

AVAILABILITY OF DATA AND MATERIALS: The datasets used and analyzed during the current study are available from the authors on reasonable request.

COMPETING INTERESTS: AJH and CF have received research funding and consultancy fees from Guanguong Shunde Innovative Design Institute, Guangdong, China. The remainder of the authors have no disclosures.

FUNDING: None.

AUTHORS CONTRIBUTIONS: AJH, MTB, and CF designed the study. AJH, AB, EB, AC, ASE, JJ, and VT performed data acquisition. AJH, MTB, XM, AML, and CF performed data analysis and interpretation. AJH and MTB drafted the manuscript. AB, EB, AC, ASE, JJ, VT, XM, AML and CF critically revised the manuscript. All authors have approved the submitted version and agree to be personally accountable for their contributions and ensure that questions related to the accuracy and integrity of the work are appropriately investigated, resolved, and the resolution documented in the literature.

ACKNOWLEDGMENTS: The authors would like to thank Ms. Adela Mas for assistance with database preparation, Ms. Elena Ramentol for assistance with data collection, and Dr. Luz María Peña-Longobardo and Dr. Ana Magdalena Vargas for their guidance with the statistical analysis.

REFERENCES

1. Zhang YN, Chen Y, Wang Y, et al. Reduction in healthcare services during the COVID-19 pandemic in China. *BMJ Glob Heal*. 2020;5(11):1-10. doi:10.1136/bmjgh-2020-003421
2. Gondi S, Chokshi DA. Financial Stability as a Goal of Payment Reform - A Lesson from COVID-19. *JAMA - J Am Med Assoc*. 2020;324(16):1596-1597. doi:10.1001/jama.2020.18343
3. Thiel CL, Eckelman M, Guido R, et al. Environmental impacts of surgical procedures: Life cycle assessment of hysterectomy in the United States. *Environ Sci Technol*. 2015;49(3):1779-1786. doi:10.1021/es504719g
4. Hessheimer AJ, Morales X, Ginestá C, et al. Where have all the appendicitis gone? patterns of urgent surgical admissions during the COVID19 pandemic. *Br J Surg*. 2020;107(11):e545-e546. doi:10.1002/bjs.11882
5. Morales X, Turrado V, de Lacy B, Hessheimer A, Fondevila C, de Lacy AM. Experience of an emergency surgery department during the pandemic caused by SARS-CoV-2 infection. *Emergencias Rev la Soc Esp Med Emergencias*. 2020;32(6):435-436. http://www.ncbi.nlm.nih.gov/pubmed/33275368
6. Curell A, Adell M, Cirera A, et al. Decline in general surgery emergencies during COVID-19 pandemic. Has its severity increased? Analysis in a large volume hospital in Europe. *J Visc Surg*. Published online 2020:19-20. doi:10.1016/j.jviscsurg.2020.11.002
7. O’Connell RM, Khan MA, Amir M, et al. The impact of COVID-19 on emergency general surgery admissions and operative volumes: A single centre experience. *Surg*. 2020;(January). doi:10.1016/j.surge.2020.09.013
8. Bugaev N, Hojman HM, Breeze JL, et al. Acute Care Surgery Service Is Essential During a Nonsurgical Catastrophic Event, the COVID-19 Pandemic. *Am Surg*. Published online 2020:000313482097208. doi:10.1177/0003134820972084
9. Baugh JJ, White BA, McEvoy D, et al. The cases not seen: Patterns of emergency department visits and procedures in the era of COVID-19. *Am J Emerg Med*.

2020;(January). doi:10.1016/j.ajem.2020.10.081

10. Boyle LI, Boyle A, Jay S, Marnewick J. COVID-19 lockdown impact on common general surgical acute presentations to a regional centre in New Zealand. *N Z Med J*. 2020;133(1525):96-105. http://www.ncbi.nlm.nih.gov/pubmed/33223552

11. Doyle DJ, Goyal A, Bansal P, Garmon EH. *American Society of Anesthesiologists Classification*.; 2020. http://www.ncbi.nlm.nih.gov/pubmed/28722969

12. Slankamenac K, Graf R, Barkun J, Puhan MA, Clavien PA. The comprehensive complication index: A novel continuous scale to measure surgical morbidity. *Ann Surg*. 2013;258(1):1-7. doi:10.1097/SLA.0b013e318296c732

13. Glick HA, Doshi JA, Sonnad SS, Polsky D, eds. *Economic Evaluation in Clinical Trials*. Second Edi. Oxford Univ; 2007.

14. Manning WG, Mullahy J. Estimating log models: To transform or not to transform? *J Health Econ*. 2001;20(4):461-494. doi:10.1016/S0167-6296(01)00086-8

15. Riquelme F, Muñoz C, Ausania F, et al. Laparoscopic versus open hemihepatectomy: comprehensive comparison of complications and costs at 90 days using a propensity method. *Updates Surg*. Published online 2020. doi:10.1007/s13304-020-00854-y

16. Staiger RD, Cimino M, Javed A, et al. The comprehensive complication index (CCI1) is a novel cost assessment tool for surgical procedures. *Ann Surg*. 2018;268(5):784-791. doi:10.1097/SLA.0000000000002902

17. Bemanian M, Mæland S, Blomhoff R, et al. Emotional eating in relation to worries and psychological distress amid the covid-19 pandemic: A population-based survey on adults in norway. *Int J Environ Res Public Health*. 2021;18(1):1-10. doi:10.3390/ijerph18010130

18. Robinson E, Gillespie S, Jones A. Weight-related lifestyle behaviours and the COVID-19 crisis: An online survey study of UK adults during social lockdown. *Obes Sci Pract*. 2020;(July):735-740. doi:10.1002/osp4.442

19. Naughton F, Ward E, Khondoker M, et al. Health behaviour change during the UK COVID-19 lockdown : Findings from the first wave of the C-19 health behaviour and

well-being daily tracker study. Published online 2021. doi:10.1111/bjhp.12500

20. Nash DB, Angelo M, Nash EJ, Gleason JL, Meyer BA. We Know Health Is Not Elective: Impacts of COVID-19. *Popul Health Manag*. 2020;23(5):378-385. doi:10.1089/pop.2020.0161

21. Chatterji P, Li Y. Effects of the COVID-19 Pandemic on Outpatient Providers in the United States. *Med Care*. 2021;59(1):58-61. doi:10.1097/MLR.0000000000001448

22. Molero García JM, Arranz Izquierdo J, Gutiérrez Pérez MI. Atención Primaria COVID-19 in Spain , how did we get here ? *Atención Primaria*. 2020;52(10):676-679. doi:10.1016/j.aprim.2020.10.003

23. Plott CF, Kachalia AB, Sharfstein JM. Unexpected Health Insurance Profits and the COVID-19 Crisis. *JAMA - J Am Med Assoc*. 2020;324(17):1713-1714. doi:10.1001/jama.2020.19925

24. Hashmi P, Fahad S, Naqi Khan H, Zahid M, Sadruddin A, Noordin S. Covid-19 pandemic: Economic burden on patients with musculoskeletal injuries in a tertiary care hospital of LMIC; retrospective cross sectional study. *Ann Med Surg*. 2020;60(July):5-8. doi:10.1016/j.amsu.2020.09.049

25. Li S, Zeng M, Dong J, et al. Management of Endovascular Treatment for Acute Ischemic Stroke During the COVID-19 Pandemic at a Single Institution in Beijing, China: A Brief Report. *J Neurosurg Anesthesiol*. Published online November 20, 2020. doi:10.1097/ANA.0000000000000748

TABLE 1. Baseline sociodemographic and clinical characteristics of patients with unplanned general surgery admissions during two periods: 02-05/2019 (pre-pandemic) and 02-05/2020 (COVID19 pandemic).

	**2019 (N=426)**	**2020 (N=346)**	***P***
Age (y)	59±20	60±20	0.55
Age range (%)			0.44
18-30	9	10	
31-40	12	10	
41-50	17	13	
51-60	13	15	
61-70	15	15	
71-80	14	19	
81-90	15	15	
>90	5	3	
Women (%)	41	45	0.24
Country of birth (%)			0.73
Spain	80	76.1	
Rest of Europe	3.5	3.2	
Middle East	0.7	1	
Africa	1.8	2.7	
Latin America	10.6	13	
Asia	2.8	2.1	
North America	0.5	0	
N/A	0.3	1.6	
Civil status (%)			0.001
Single/no partner	16.4	10.7	
Married/couple	40.6	36.6	
Divorced/separated	5.9	6	
Widow/-er	9.4	5.2	
N/A	27.7	41.5	
Level of education (%)			0.10
Illiterate/unable to read or write	2	1	
Incomplete primary education	9.4	8	
Complete primary education	26.8	22	
Secondary education	10.4	15	
High school diploma or equivalent	14.1	11.5	
Associate’s degree	9.1	11.5	
Bachelor’s degree	24.5	30.5	
Postgraduate education	3.7	0.5	
Employment situation (%)			0.32
Employed	37	36.1	
Pensioner who has worked	45.7	47.3	
Pensioner who has not worked	5.7	2.9	
Unemployed	5.3	8.8	
Student	2	1	
Unpaid domestic work	2.3	1.5	
Other	0.7	1.9	
N/A	1.3	0.5	
Profession (%)			0.50
Director, manager, or administrator	4	1.6	
Scientific professional, intellectual	23.5	29	
Technician, support professional	18	18.1	
Accountant, office worker	1.8	3.6	
Food service worker, personal care provider, protection, or sales	20	20.2	

Skilled worker in agriculture, livestock, forestry, or fishing	0.4	0	
Skilled craftsperson, manufacturing, or construction worker	16.9	16.1	
Installation, machinery, or assembly worker	3.3	3.1	
Elementary or secondary education provider	12.1	8.3	
ASA classification (%)			0.47
I	23.7	25	
II	52.8	48.5	
III	21.1	25	
IV	2.4	1.5	
Previous surgery (%)	42.5	38.8	0.29

Continuous variables are presented as mean ± standard deviation and categorical variables as frequencies. ASA, American Society of Anesthesiologists; N/A, information not available.

TABLE 2. Variables related to admission and management and outcomes of hospitalization for patients with unplanned general surgery admissions during two periods: 02-05/2019 (pre-pandemic) and 02-05/2020 (COVID19 pandemic).

	**2019 (N=426)**	**2020 (N=346)**	***P***
Admission			
Diagnostic category			0.01
Appendicitis	106 (24.9)	71 (20.6)	
Biliary tract pathology	89 (20.9)	103 (29.8)	
Gastrointestinal obstruction	73 (17.1)	49 (14.2)	
Non-obstructive gastrointestinal pathology	85 (20)	53 (15.4)	
Skin & soft tissue infection	21 (4.9)	25 (7.2)	
Abdominal trauma	12 (2.8)	3 (0.9)	
Readmission	31 (7.3)	28 (8.1)	
Other	9 (2.1)	13 (3.8)	
Onset of symptoms to diagnosis (days)	2±3	5±22	0.01
Non-operative management	174 (41)	150 (43)	0.43
Failure of non-operative management	11 (7)	18 (11)	0.22
Surgical intervention			
Intraoperative complications	8 (2)	5 (1)	0.64
Operative time (min)	89±93	92±59	0.63
Hospitalization & outcomes			
ICU stay (days)	0.9±5	0.6±3	0.40
Overall hospital stay (days)	9±16	7±11	0.19
Surgical reintervention	12 (3)	8 (2)	0.66
CCI	10.3±23.7	13.9±25.5	0.10
Mortality	13 (3)	13 (4)	0.59

Continuous variables are presented as mean ± standard deviation and categorical variables as "N" (%). CCI, Comprehensive Complication Index; ICU, intensive care unit.

TABLE 3.Unadjusted and adjusted healthcare costs (2020 €) for patients with unplanned general surgery admissions during two periods: 02-05/2019 (pre-pandemic) and 02-05/2020 (COVID19 pandemic).

	Unadjusted							Adjusted				
	2019 (N=409)			2020 (N=334)				2019		2020		
	**Mean**	**SD**	**Median**	**Mean**	**SD**	**Median**	***P***	**Mean**	**SE**	**Mean**	**SE**	***P***
Total hospital-associated healthcare costs[1,2]	5,886.29	12,576.33	2,535.77	5,287.62	7,220.16	3,217.4	0.43	4,656.89	390.53	4,938.54	406.55	0.28
Hospitalization costs												
Pharmacy[1,3]	401.16	2,034.31	22.89	241.76	877.82	37.73	0.17	177.98	79.06	184.77	88.10	**0.04**
Transfusions[4]	54.95	302.57	0	40.66	210.75	0	0.46	53.83	13.15	41.34	10.54	0.62
Artificial implants[3]	63.93	356.76	0	81.85	376.48	0	0.50	58.33	14.30	67.24	21.24	0.51
Miscellaneous consumable materials[3]	69.36	371.22	0	172.30	502.56	0	**0.001**	82.57	28.64	190.78	32.69	**0.02**
Physician medical attention [3]	380.27	1082.06	154.17	359.07	562.51	184.26	0.74	225.57	39.88	284.64	25.66	0.91
General floor stay[3]	2,319.95	5,236.91	866.01	2,021.07	3,326.40	992.45	0.36	1,444.76	238.67	1,485.71	148.55	0.34
Studies and procedures[3]	309.09	890.36	0	335.66	834.68	0	0.67	331.57	50.32	264.13	42.54	0.29
Laboratory tests[2]	85.78	277.83	13.17	79.16	150.31	25.76	0.69	56.12	9.44	70.21	10.60	0.48
Specialist consultations[2]	97.72	199.36	0	101.13	162.34	0	0.80	74.49	7.17	89.41	11.23	0.16
Sanitation/janitorial services[3]	150.51	267.76	64.50	127.99	169.80	73.72	0.17	100.54	12.67	99.90	8.17	0.09
Surgical costs												
OR staff[3]	567.56	857.54	459.9	549.67	736.83	471.75	0.76	569.09	38.33	618.18	43.31	0.70
OR usage[3]	755.91	1,099.05	698.42	635.85	867.52	607.52	0.09	755.87	47.59	715.59	46.18	0.07
OR consumables[3]	200.44	409.52	198.11	153.56	180.11	198.11	**0.04**	199.02	17.34	169.73	9.47	**0.01**

ASA, American Society of Anesthesiologists; OR, operating room; SD, standard deviation; SE, standard error.
[1]Ajdusted costs calculated using a generalized linear model (GLM). All other costs were calculated using a two-part model, with GLM in the second part of the model.
[2]Adjusted costs adjusted by age, level of education, profession, ASA classification, and history of previous surgery.
[3]Adjusted costs adjusted by age, ASA classification, and history of previous surgery, based on collinearity.
[4]Adjusted costs adjusted by age and history of previous surgery.

FIGURE LEGENDS.

FIGURE 1. Monthly evolution of unplanned general surgery consults and admissions during two periods: 02-05/2019 (pre-pandemic) and 02-05/2020 (COVID19 pandemic).

FIGURE 2. Monthly evolution of total per-person healthcare costs for unplanned general surgery admissions during two periods: 02-05/2019 (pre-pandemic) and 02-05/2020 (COVID19 pandemic).

FIGURE 1.

Click here to access/download;Figure;FIGURE_1.pdf

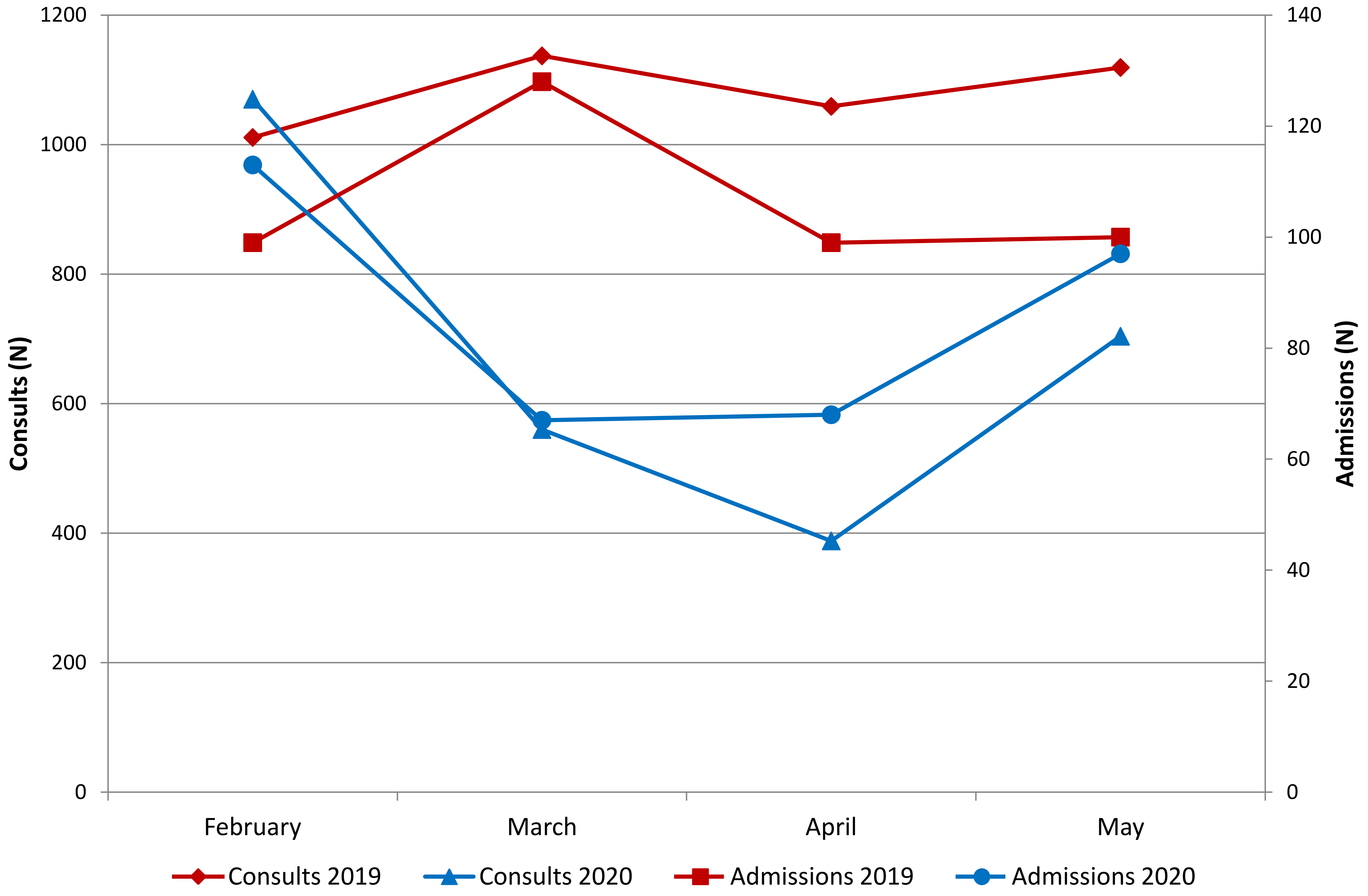

FIGURE 2.

Click here to access/download;Figure;TOTALCOSTS_monthly.tif

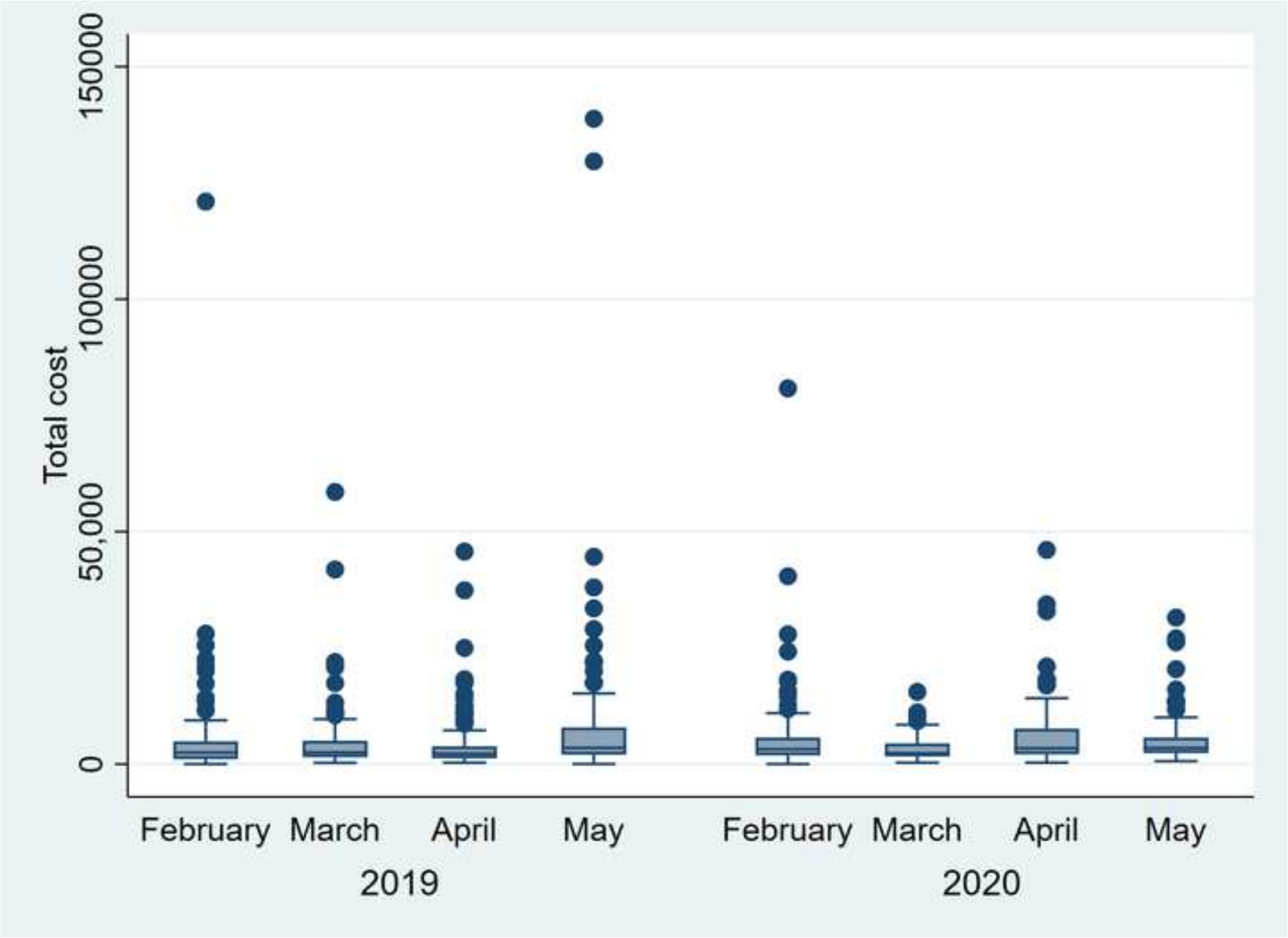

Supplement: Attachment — Submitted filename: PONE-D-21-12253_reviewer.pdf [file pone.0252919.s001.pdf]
